# Interactive 3D Force/Torque Parameter Acquisition and Correlation Identification during Primary Trocar Insertion in Laparoscopic Abdominal Surgery: 5 Cases

**DOI:** 10.3390/s22228970

**Published:** 2022-11-19

**Authors:** Nantida Nillahoot, Branesh M. Pillai, Bibhu Sharma, Chumpon Wilasrusmee, Jackrit Suthakorn

**Affiliations:** 1 Department of Biomedical Engineering, Center for Biomedical and Robotics Technology (BART LAB), Faculty of Engineering, Mahidol University, Nakhon Pathom 73170, Thailand; 2 Department of Surgery, Faculty of Medicine Ramathibodi Hospital, Mahidol University, Bangkok 10400, Thailand

**Keywords:** force/torque data acquisition, laparoscopic surgery, minimally invasive surgery, medical robotics, robotic surgery

## Abstract

Laparoscopic procedures have become indispensable in gastrointestinal surgery. As a minimally invasive process, it begins with primary trocar insertion. However, this step poses the threat of injuries to the gastrointestinal tract and blood vessels. As such, the comprehension of the insertion process is crucial to the development of robotic-assisted/automated surgeries. To sustain robotic development, this research aims to study the interactive force/torque (F/T) behavior between the trocar and the abdomen during the trocar insertion process. For force/torque (F/T) data acquisition, a trocar interfaced with a six-axis F/T sensor was used by surgeons for the insertion. The study was conducted during five abdominal hernia surgical cases in the Department of Surgery, Faculty of Medicine, Ramathibodi Hospital, Mahidol University. The real-time F/T data were further processed and analyzed. The fluctuation in the force/torque (F/T) parameter was significant, with peak force ranging from 16.83 N to 61.86 N and peak torque ranging from 0.552 Nm to 1.76 Nm. The force parameter was observed to positively correlate with procedural time, while torque was found to be negatively correlated. Although during the process a surgeon applied force and torque in multiple axes, for a robotic system, the push and turn motion in a single axis was observed to be sufficient. For minimal tissue damage in less procedural time, a system with low push force and high torque was observed to be advantageous. These understandings will eventually benefit the development of computer-assisted or robotics technology to improve the outcome of the primary trocar insertion procedure.

## 1. Introduction

With minimum postoperative pain, faster recovery time, and an enhancement in the overall quality of treatment, minimally invasive surgery (MIS) has become a standard choice of treatment for abdominal surgeries [1]. The advent of the notion has substantially eased procedures such as cholecystectomy, gastric ulcer surgery, colon surgery, splenectomy, and surgeries regarding inguinal and ventral hernias [2]. Considering the scenario for the past decade, growth has seemed momentous, with a high percentage of surgeries being performed along this paradigm [3]. Generally, the procedure begins with the primary trocar insertion, giving first exposure to the surgical workspace inside the abdominal cavity. Primary trocar insertion is a skill-demanding task that requires experienced surgeon participation in order to achieve preferred outcomes [4]. However, the most common intraoperative complications are usually associated with trocar insertion [5,6,7,8].

On the one hand, there are a plethora of advantages associated with MIS; on the other, advancement in technology is demanded for its further adoption. In this light, robotic or robot-assisted surgery has been able to garner significant attention [9,10]. Understandingly, this has induced the prolific growth of robotic surgery and computer assisted surgery, with growing acceptance [11,12,13,14]. However, regarding acceptance, robotic surgery still encounters challenges posed mainly due to cost, operation, safety, and effectiveness [15,16]. Taking this concern into consideration, the MU-Laparobot project has been developed for computer-assisted MIS under the platform of robotics surgery. One of the aims is to develop an assistive system for the limiting of surgical tool force in primary trocar insertion [17,18]. Moreover, force limiting is crucial for both the manual as well as robotic trocar insertion, as excess axial force is responsible for injuries in the patient [19,20]. Although the study was successful in terms of control system development, data regarding external force in practical situations for a bilaterally controlled console were limited. The purpose of this study is to collect and analyze the force and torque characteristics as the surgeon conducts the primary trocar insertion process. The analysis underlines force–torque responses that signify or reflect the character of each layer of abdominal layers, which include the epidermis, dermis, subcutaneous tissue, linea alba fascia, preperitoneal fats, and parietal peritoneum.

To substantiate the research objective, data acquisition was conducted in real-time surgeries and analyzed accordingly. The components of the research are reflected in the organization of the paper. While Section 2 discusses related bodies of the literature review, Section 3 delineates the tools, materials, and proposed method to conduct the experiment and the data analysis. Similarly, Section 4 illustrates the results from the study and Section 5 consists of the summary and the analysis of the dynamics involved with the insertion process. This involves tabulated data as well as interactive graphs for enhanced comprehension. Finally, critical analysis with respect to the prevailing literature and the summary as a conclusion is described in Section 6.

## 2. Related Works

The incorporation of primary trocar insertion function in a robotic system is an intricate task. The availability of the data pertaining to the insertion process is scarce, including the interactive force/torque parameters. Most of previous studies had been performed in laboratory settings of nonhuman tissue samples [21,22,23,24,25]. The study involving real surgical cases or soft human tissues (cadavers) did not sufficiently discuss the quantitative analysis of the acquired data [26]. Moreover, in a survey performed by Gerwen et al. in [27], where they reviewed 107 papers regarding the study of force in needle–tissue interaction, a few studies were associated with the experiment on live tissue. Interestingly, none of the reviewed papers reported real-time surgical data. Most of the related force studies have been performed on porcine skin, which resembles human tissue [28,29,30] and abdomen phantoms [31]. Furthermore, another aspect of studying the force characteristics of the interaction has been through the estimation process. In [22], a sensorless bilateral operation was illustrated that considers the force estimation for force control. Similarly, [21] implemented a neural network to estimate interactive torque and verified it through an experimental procedure.

Similarly, in other surveys, the tool–tissue interactive force for various types of surgeries was investigated during several procedures such as penetration, retraction, grasping, dissection, clamping, and cutting [32,33]. General surgery involving the laparoscopic procedure was observed to have highest mean average interactive force [34,35]. However, in 14 studies involving general surgery in laparoscopic, conventional, and robotic methods, none of the studies involved data acquisition in a real surgical scenario. Moreover, despite the laparoscopic procedure being discussed, the process of trocar estimation, which involves an intricate force/torque response, was not studied in any of the studies. With a thorough review of the literature, the lack of a real-time interactive force/torque dataset from an abdominal surgery was evident. However, despite information from phantoms and animal skin, the design and control of such a robotic system would face impediments without kinetic data from the real scenario [36,37,38,39]. Therefore, this research primarily focuses on the acquisition of real-time interactive force/torque data from a real surgical procedure. Most of the previous studies had been performed on laboratory settings of nonhuman tissue samples. The studies on real surgical cases or soft human tissues were also limited in term of quantitative analyses [23,25,40,41,42]. These understandings will eventually benefit the development of computer-assisted or robotics automation technology to improve the outcome of the procedure [10,43,44,45,46].

## 3. Materials and Methods

Abdominal hernia cases that were designated to receive laparoscopic surgery for repair treatment were selected from the Department of Surgery, Faculty of Medicine, Ramathibodi Hospital, Mahidol University. All cases were considered as obese abdominal contouring according to the surgeon’s opinion. There was no specification of surgeon’s experiences or specific qualifications—surgical residence doctors under supervision were also applicable in our study; all principal surgeons who performed the task differed from case to case. The specification of the trocar is shown in Table 1.

**Figure 1:**
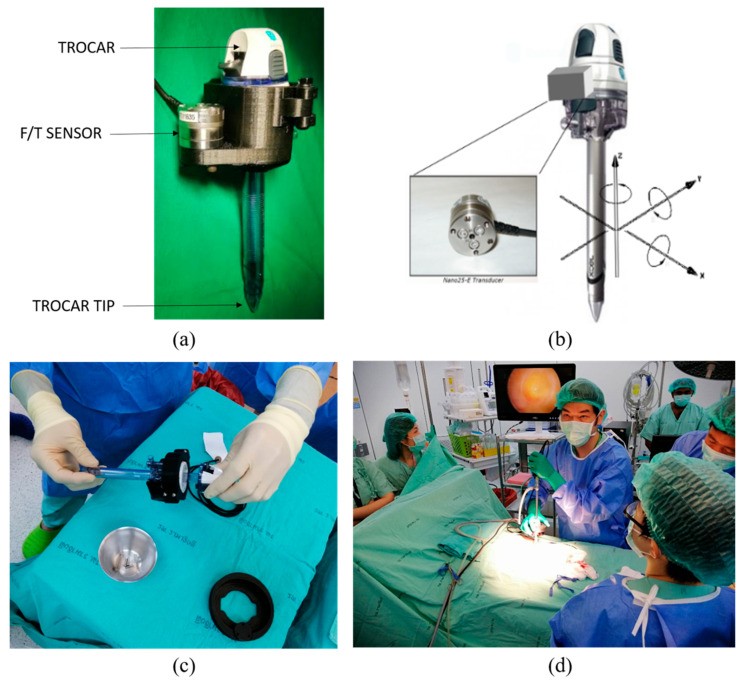
(**a**) Shows the trocar XCEL endopath size 12 and F/T sensor Nano25 from Ati; (**b**) orientation of coordinates for dynamics description; (**c**) surgeon assembling the modified F/T module; (**d**) laparoscopic primary trocar insertion with the attached modified F/T module.

To record force–torque characteristics, F/T sensor ATi Nano25 was used for measurement of 3 axes of force (Fx, Fy, Fz) and 3 axes of torque (Tx, Ty, Tz); orientation was as Figure 1. The sensor can measure the maximum axial load of 500 N at 1/16 N resolution. Similarly, the maximum torque of 3 Nm can be sensed at a resolution of 1/2640 Nm. The sensor was initially calibrated by manufacturer ATi complying with the National Institute of Standards and Technology (NIST) and can be reconfigured and recalibrated using the available driver software during the initialization of the system. The sampling frequency for the sensor system is set at maximum by default and is prescribed to change only for special circumstances. Therefore, the experiment was conducted at 1000 Hz.

The sensor was mounted onto a custom-designed 3D-printed holder acting as a case for the trocar. Polylactic acid (PLA) was selected as the material for 3D printing as it demonstrates high biocompatibility [47]. Moreover, all the components excluding the surgical tools, including mounting, sensor, and wiring, were enclosed with sterilized polythene before each of the procedures. The 3D-printed parts also include mounting parts for surgeon’s better grips. As it was observed that the palm is the primary source of force during trocar insertion, the mounting location of the force sensor was selected so that the force originating from the palm could be directly acquired. Moreover, directly placing the sensor below the palm does not generate moment of force, and the entirety of the measured force can be comprehended as the compressive load on the sensing body. The sensor was connected through digital converter to computer for recording data. The recording frequency was set to 1000 Hz. Investigators started a recording session when surgical field was prepared, and a principal surgeon began to insert trocar into cadaver’s abdomen. The location of insertion was at linea alba, just below the umbilicus. The sensor recorded the force and torque according to time until the trocar passed through peritoneum layer into abdominal cavity. Recorded datasets were imported into MATLAB 2019b for analysis and visualization. A quantitative summary of force–torque according to time of each case was performed. Modifications to the data were performed for better visualization, which are described in the results’ figures. This study was approved by Human Research Ethics Committee, Faculty of Medicine Ramathibodi Hospital, Mahidol University, IRB No (COA. MURA2022/110). Informed consent was obtained from all subjects and all methods were performed in accordance with the relevant guidelines and regulations.

## 4. Results

### Cases Summary

The data from five abdominal hernia cases of soft human tissues were collected, as summarized in Table 2. These data can be described as follows: For the character of the exerted force, the maximum amount used for each case was: 61.86 N, 16.83 N, 29.22 N, 19.16 N, 28.60 N. For the character of the torque, the maximum amount measured for each case was: 1.76 Nm, 0.552 Nm, 0.721 Nm, 0.629 Nm, 1.11 Nm. The most prominent axis that contains major dynamics data of the procedure is the z-axis. This is due to the nature of positioning the trocar insertion direction, which mostly involves vertical movement. Ideally, this action can be perfectly performed as a linear single-dimension motion; however, this cannot be achieved practically by any surgeon. The first case seems to be an outliner, which resulted due to the exceeding duration of action when compared to others. Furthermore, it was found that this case exhibited the maximum amount of force (61.86 N) with the longest pause time. These characteristics signify struggles of the surgeon to put the trocar through abdominal wall layers. For other cases, the range of the maximum force exerted was varied from 12.39 N to 16.83 N on the second case and up to 29.22 N on the third case. Average force exerted by excluding the first case ranged from 9.78 N on the second case and up to 17.47 N on the fifth case.

**Figure 2:**
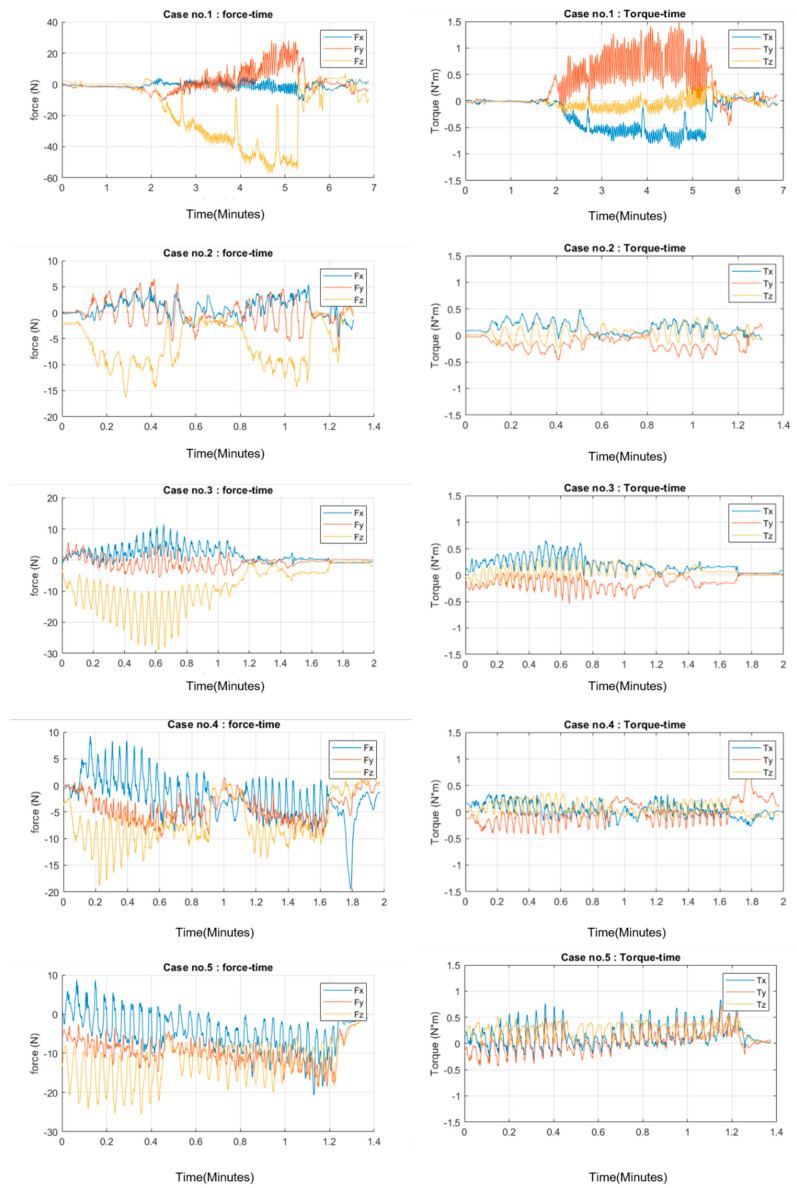
Three–dimensional force–torque characteristics’ representation through time of the primary trocar insertion procedure in all 5 cases. Fx: force on x–axis, Fy: force on y–axis; Fz: force on z–axis; Tx: torque on x–axis; Ty: torque on y–axis; Tz: torque on z–axis; N: newton, m: meter.

These data show a wide range of force level profiles used to accomplish the procedure. The factors related come from both the surgeon’s technique and the patient’s anatomical structure variations. However, the objective of the study is to observe the pattern of dynamics that might occur and not the exact value of quantitative similarities. Therefore, the graph of each dataset to observe its relation to time flow through procedure is as follows (Figure 2).

## 5. Discussion

### 5.1. Dynamics of Primary Trocar Insertion

**Figure 3:**
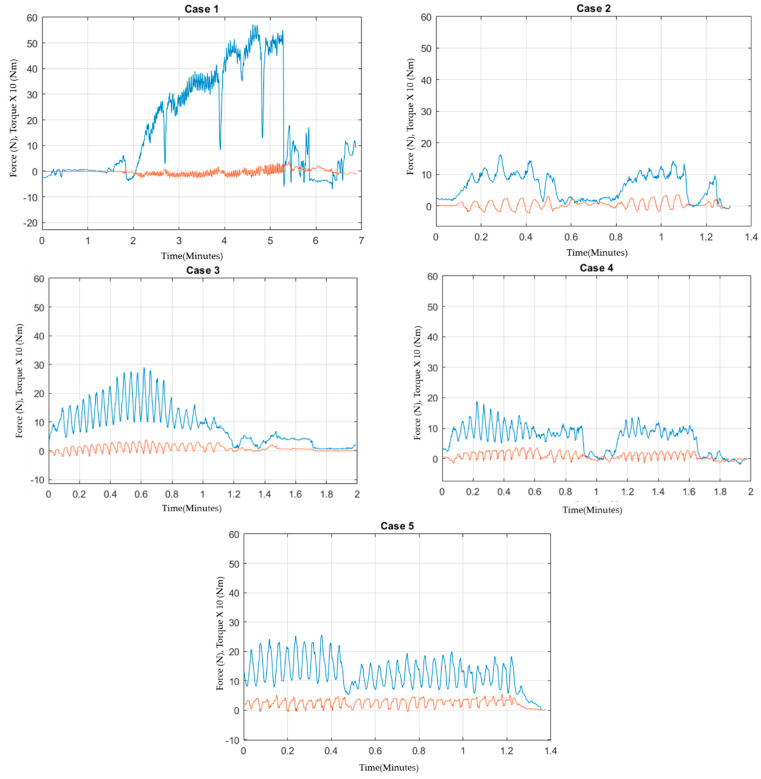
Force−torque characteristics through time of the procedure by focusing on Fz (blue) and Tz (orange). Fz and Tz were main representors for the dynamics of trocar insertion. It is noted that the magnitude of torque was multiplied for better visualization.

Figure 3 show the force and torque data collected from each case. These figures are based on data that were collected for further analysis of the study. The characteristics of each surgeon’s performance and trocar dynamics are discussed in detail. Figure 3 highlights the key motion of trocar insertion as performed by surgeons for all five cases. The reason we chose the z-axis data as our main representation of the dynamics of primary trocar insertion is because of the procedure settings and preferred orientation, as mentioned in previous section.

**Figure 4:**
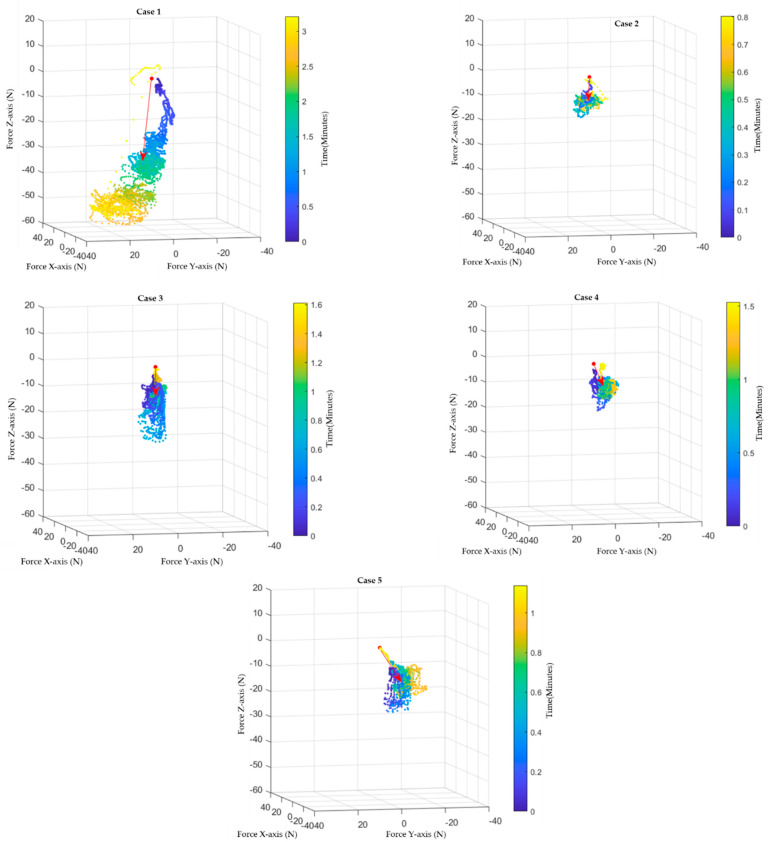
Scatter plots and vector plots of each case are shown. The average force vector is shown by the red arrow of each figure. The scatter plots describe each vector point of force and are marked by different colors to illustrate time flows (blue to yellow: early to late phase of action).

Figure 4 illustrates the scatter plots of resultant force vectors related to time in the XYZ coordinate vector space and the average resultant force of each case. Interpretations of each case are as follows: For the first case, the amount of force exerted increased in relation with time. The sharp-rising steady increase followed by the abrupt breakdown signifies the point of breaking through the peritoneum. Even though this pattern signifies the increase in overall elastic resistance proportional to the surgeon’s effort, it did not exhibit any differences in the elastic resistance of each specific layer. In this case, the surgeon also exerted a small amount of torque around the z-axis compared to vertical force exertion, which means that the surgeon was trying to fight back the resistive property of tissues by pushing straight with ineffective rotary motion.

Moreover, from Figure 4, we can see that the first case shows the highest variation in force direction. Although the average resultant vector of the force was the most aligned with the z-axis, this came from the high amount of force used rather than the accurate point control of the instrument, as shown from variation in direction. Therefore, this might be the dataset that shows an improper technique, which results in longer time to perform, with a significantly higher amount of force. As the magnitude of force increases, it paves the way for more tissue injuries and a longer recovery time. Hence, we can conclude that the primary trocar insertion had the poorest outcome for the first case.

In second case, the force characteristics had steady contours throughout the procedure. Fluctuations at the contour apex occurred because of the periodic pushes of the surgeon. The silent dynamics period at the middle of the action was a result of the pause by the surgeon during performing the procedure. Again, because of the difference in force level throughout the procedure, we cannot interpret any differences between each elastic soft tissue. However, this dataset still gives detailed suggestions for a better type of dynamics for trocar insertion. From the torque perspective, the relation to force exerted was aligned. This means that the pushing and rotation of the trocars as one smooth motion helps in decreasing the amount of force required to perform the procedure with a minimal degree of injuries to the tissue. This type of dynamic profile was also observed in case number 4 and 5; variations between them were only the level of force and the frequency of the pushing–rotating motion, which was more likely related to the patient’s anatomical factors. For the third case, the pattern of force was different from the two main groups mentioned above. The synchronization between pushing and rotating dynamics persisted. However, the amount of exerted force was unique. There was an ascending–descending pattern with smooth changes in force level contour. Comparatively, the character might be considered to fall in between the two groups mentioned before. This dataset might give us a clue to locate how soft tissue layers related to the amount of force.

The amount of force required to penetrate elastic tissue should be higher in the layer of dermis than the later layer of subcutaneous and preperitoneal fats. However, these data may just occur from the surgeon exerting more force than is required. Then, the surgeon decreased the amount of force as he was precautious approaching the peritoneum. Further collection of data is required before making an assumption into any of these two hypotheses. As a result, we can conclude that a common pattern of motion is a rotating (torque acting around the z-axis) and pushing motion (force acting on the z-axis). The spikes of force may indicate a stroking motion, as the surgeon periodically pushed and twisted the trocar through layers of tissue. We also observed from these figures that the first case has the lowest ratio of torque to force on the z-axis. This supports that the motion this surgeon performed was driven by exerting force onto the z-axis more than rotating the trocar during the pushing action.

### 5.2. Relationship to Depth of Penetration

**Figure 5:**
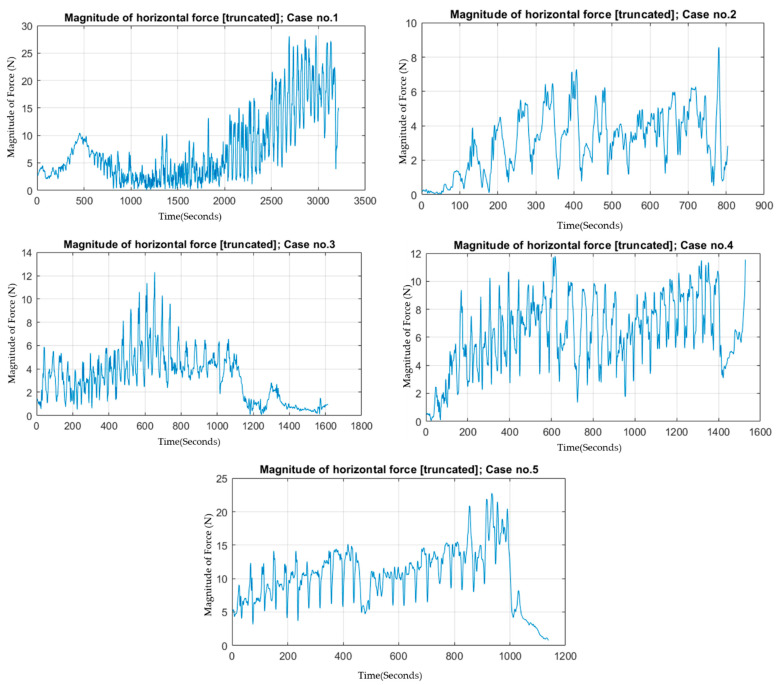
Magnitude of horizontal force including force on x-axis and y-axis according to time of action of each case.

We further investigated the aspect of how we can interpret or determine force–torque characteristics that can specify the layers of abdominal walls. As the trocar penetrated through abdominal walls, frictional force increased proportionally with surface contact between the trocar and contacted tissues. This resulted in increased compressive resistance in motion disturbances in the horizontal plan (xy-axes). Therefore, the amount of force exerted to overcome this friction in order to create an adequate amount of torque and rotational motion should be increased. In order to test if this hypothesis can be applied in practical observation or not, Figure 5 is designed to show the magnitude of horizontal force according to time. From Figure 5, case 1, case 4, and case 5, we can observe the mentioned behavior as the horizontal force increases with respect to time. However, case 2 and case 3 do not exhibit this trend.

**Figure 6:**
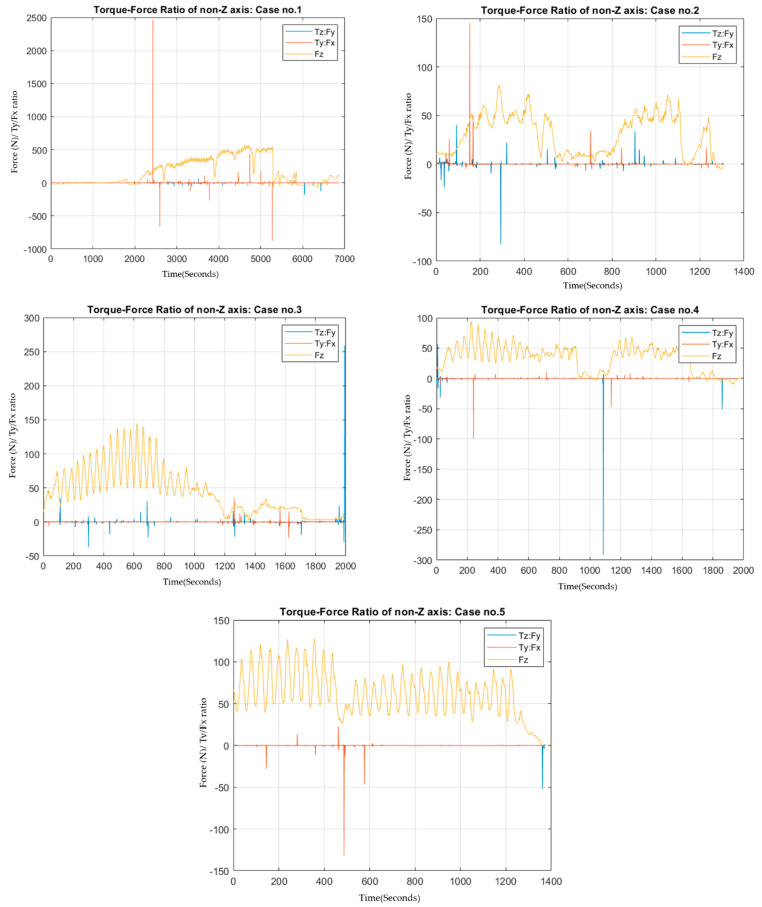
Force on z−axis according to time with torque to force ratio on non−z−axes through time. The magnitude of force was multiplied for better visualization.

Regarding the fictitious force effect and positioning of the trocar of specific location, we suspected that if the surgeon tried to perform a rotating motion of the trocar while pushing through tissues, the further the trocar traveled, changes would occur in the torque ratio compared to the amount of exerted force. Therefore, the plotting of the T: F ratio on the non-z-axes might give us a clue of layer penetration (Figure 6).

From Figure 6, these spikes occurred from sudden changes in nonvertical torque-to-force ratio. As mentioned, we hypothesize that the depth that the trocar penetrates should affect how the torque manipulation reacts to horizontal force. However, we cannot directly illustrate this relationship with acquired information as the torque axis used was located at the trocar grip, not the point of contact between the tissue and non-blade-end point of the trocar. Still, these illustrations might give us a clue to evaluate the possibility of this hypothesis for indirect depth calculation. These spikes are located at the point of important dynamics changes.

The first type of dynamics changes that we can confirm from observation is the pausing point and redoing point. Secondly, there are high spikes comparable to the magnitude of the first type of spikes. These spikes might relate to the layer breakthrough as the trocar penetrates the epidermis to the dermis and from the dermis into the subcutaneous layers and so on. Third, there are random low-value spikes, for which we have no explanation of what they might represent. These assumptions are not in any way valid, but are only suggestions for further improvements of the study design or applications to further tests/data collection designs, which may give us more reliable and viable data for this type of analysis [48].

### 5.3. Correlation Analysis

**Figure 7:**
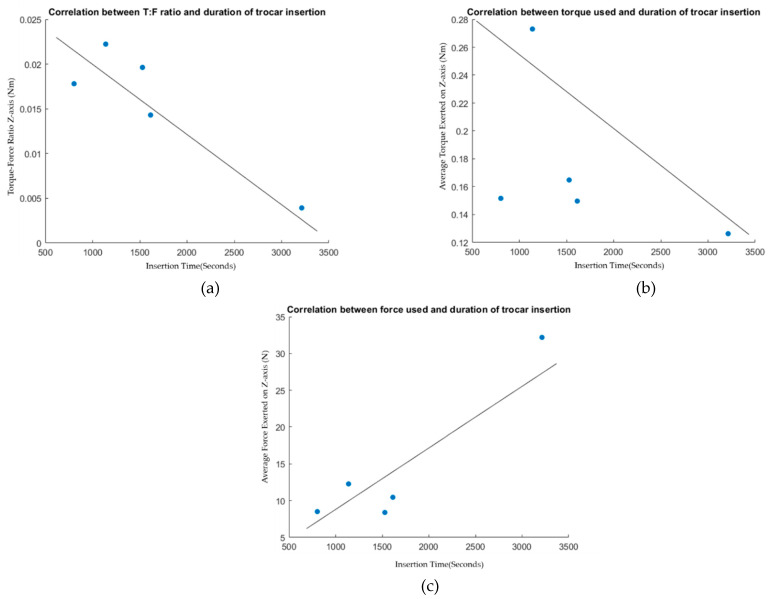
Scatter plots show correlation between force and torque factors to procedural outcome and time to perform the action. (**a**) Shows correlation between T: F and duration of trocar insertion; (**b**) shows correlation between torque used and trocar insertion time; (**c**) shows correlation between force exerted during procedure and procedural time.

As suggested by evidence shown in summary Table 1, the preferred outcome of primary trocar insertion might be predictively determined by average exerted force and torque, and more interestingly, the ratio between the two. Therefore, statistical correlation tests were carried out on to evaluate three pairs of data: Figure 7a—correlation between T: F and duration of trocar insertion; Figure 7b—correlation between torque used and trocar insertion time; Figure 7c—correlation between force exerted during procedure and procedural time. The result was as follows: negative correlation in T: F and time (R = −0.9067), poor negative correlation in torque time (R = −0.4881), highly correlated positive force time (R = 0.9289). This helps to signify that lowering the force with high torque ratio is the way to achieve an optimal time to perform the procedure.

## 6. Conclusions

The objective of the study was to comprehend the primary trocar insertion process from the perspective of kinetic parameters. The goal is to contribute towards the development of a surgical robot that can successfully perform the task. In summary, this study shows that amount of force required for trocar insertion has large variation. The variation was caused by the surgeon’s technique and experiences, as well as the patient’s anatomy. The most common and effective technique demonstrated in this study was synchronization between pushing and rotating the trocar. This technique was shown to benefit in reducing the amount of vertical force exertion and the average force acting on the tissue throughout the procedures. Time to perform was significantly negatively correlated with vertical torque–force ratio; therefore, a good powered rotating motion should outperform only a straight pushing motion and result in minimal tissue damage due to the reduced amount of force required to overcome the elastic property of the tissue. Further data collection is required to make an assumption on the elation of force–torque characteristics to the depth of tissue layer penetrated. The patterns were varied into many groups as described; therefore, more datasets should reveal the hierarchy of the majority of techniques or patterns effectively performed among surgeons. However, the other implication of the study regarding the fulfilment of the research objective is that the study is pivotal to serve as a prescriptive source for surgical robots. Based on the correlation analysis of Figure 7, the application of a high amount of torque despite the speed of procedure can successfully etch and insert the trocar with respect to the axis of insertion. This means that a robotic end-effector comprised of one axial rotation and one axial translation motion could suffice the insertion process given that the end-effector torque is high enough. Considering the average maximum torque of 11 N observed during general surgery in [32,33,34,35], the force variation observed in the trocar insertion process is below the maximum value. This means that the proposed end-effector system can comply with the requirement of a robotic system that can perform multiple actions during the general surgical procedure.

## Figures and Tables

**Table 1:** Specification of equipment used in this study.

**Surgical Trocar**	
Model	Ethicon endo surgery system XCEL endopath
specification	Size 12 (P92k16)
**Force/Torque sensor**	
Model	F/T sensor ATi Nano25
specification	Fx, Fy, Fz, Tx, Ty, Tz; frequency: 1000/s
	Maximum Fz: 500 N, maximum Tz: 3 NmForce resolution: 1/16 N, torque resolution: 1/2640 Nm

**Table 2:** Shows summary of data collected from force–torque sensor measurement during primary trocar insertion for abdominal hernia cases at Department of Surgery, Faculty of Medicine, Ramathibodi Hospital. Red-pink text: maximum value of the row. Blue text: minimum value of the row.

	Case 1	Case 2	Case 3	Case 4	Case 5
Trocar location	Midline at the base of umbilicus				
Operation	Abdominal herniation repair				
Time (unit) With pauses Without pauses Pause time	4490 3213 1277	1273 804 469	1714 1614 100	1741 1528 213	1294 1139 155
Maximum F_R_ (N)	61.86	16.83	29.22	19.16	28.60
Mean F_R_ (N)	37.22	9.78	12.75	11.27	17.47
Maximum T_R_ (Nm)	1.76	0.552	0.721	0.629	1.11
Mean T_R_ (Nm)	0.923	0.348	0.359	0.284	0.410
**T/F x-axis (Nm/N)**					
Max (N)	−10.0834	5.3399	11.4816	−11.2557	−18.4729
Mean (N)	−0.3240	1.5211	2.1746	−1.7023	−3.6505
Max (Nm)	−0.9096	0.4929	0.6544	0.3456	0.8375
Mean (Nm)	0.5549	0.2158	0.2613	0.1423	0.2375
Ratio (m)	−1.7125	0.1419	0.1202	−0.0836	−0.0651
**T/F y-axis (Nm/N)**					
Max (N)	28.2760	−7.3552	5.6133	−9.8137	−18.6518
Mean (N)	3.8928	0.3215	−0.9310	−4.9831	−8.8952
Max (Nm)	1.5168	−0.4707	−0.5462	0.6059	0.7249
Mean (Nm)	0.7274	0.2271	0.1969	0.1832	0.1930
Ratio (m)	0.1868	0.7062	−0.2115	−0.0368	−0.0217
**T/F z-axis (Nm/N)**					
Max (N)	−57.3079	−16.2875	−28.9105	−18.8188	−25.5875
Mean (N)	−32.1967	−8.5141	−10.4603	−8.3956	−12.2807
Max (Nm)	0.3427	0.3534	0.3793	0.3620	0.5395
Mean (Nm)	0.1263	0.1516	0.1497	0.1648	0.2731
Ratio (m)	−0.0039	−0.0178	−0.0143	−0.0196	−0.0222

## Data Availability

The data used to support the findings of this study are available from the corresponding author upon request.

## Abbreviations

The following abbreviations are used in the manuscript:

F/T: force/torque; MIS: minimum invasive surgery; NIST: National Institute of Standard and Technology
